# A phase I pilot study evaluating the beneficial effects of black raspberries in patients with Barrett’s esophagus

**DOI:** 10.18632/oncotarget.10457

**Published:** 2016-07-07

**Authors:** Laura A. Kresty, John J. Fromkes, Wendy L. Frankel, Cynthia D. Hammond, Navindra P. Seeram, Maureen Baird, Gary D. Stoner

**Affiliations:** ^1^ Division of Hematology & Oncology, Department of Medicine, Medical College of Wisconsin, Milwaukee, Wisconsin, USA; ^2^ Department of Internal Medicine, The Ohio State University, Columbus, Ohio, USA; ^3^ Department of Pathology, The Ohio State University, Columbus, Ohio, USA; ^4^ Department of Biomedical and Pharmaceutical Sciences, College of Pharmacy, University of Rhode Island, Kingston, Rhode Island, USA

**Keywords:** Barrett's esophagus, black raspberry, esophageal adenocarcinoma, chemoprevention, oxidative stress

## Abstract

Black raspberries inhibit a broad range of cancers in preclinical models which has led to clinical evaluations targeting premalignant lesions of the colon, oral cavity and esophagus. A phase I pilot study was conducted in twenty Barrett's esophagus (BE) patients to investigate the effect of lyophilized black raspberries (LBR) on urinary metabolites and markers of lipid peroxidation, DNA damage and tissue markers of cellular proliferation, detoxification, and inflammation. Surveys, biopsies, blood and urine samples were collected before and after 6 months of LBR treatment (32 or 45 g). LBR significantly reduced urinary excretion of 8-epi-prostaglandin F2α, a marker of lipid peroxidation linked to oxidative stress and free radical damage. Urinary levels of the ellagitannin metabolites, urolithin A-glucuronide, urolithin A-sulfate and dimethylellagic acid glucuronide were significantly increased following 12 and 26 weeks of LBR consumption and may prove useful as indicators of compliance in future clinical studies. Immunohistochemical staining of BE biopsies following LBR treatment showed significant increases in mean GST-pi levels, with 55.6% of subjects responding favorably. In summary, LBR significantly decreased urinary lipid peroxidation levels and significantly increased GST-pi, a marker of detoxification, in BE epithelium. Still, LBR may need to be formulated differently, administered at higher concentrations or multiple times a day to increase efficacy.

## INTRODUCTION

Esophageal adenocarcinoma (EAC) represents a growing health problem characterized by rapidly rising incidence, substantial morbidity and high mortality. Over the last 40 years EAC rates have increased seven-fold in the US [1] leading to EAC being the major histologic subtype of esophageal cancer in the US [2, 3]. Increasing rates of EAC and the only known precursor lesion, Barrett's esophagus (BE) are linked to persistent, symptomatic, reflux of gastric and duodenal contents, known as gastroesophageal reflux disease (GERD) [4, 5]. Heartburn is the primary symptom of GERD and is estimated to impact over 60 million Americans [4]. More recently, obesity has emerged as a strong risk factor imparting a 2 to 4-fold increase in risk for EAC [5–8] and a 1.5 to 2-fold increase in risk for GERD [5]. Furthermore, studies support a linkage between BE and obesity, especially central adiposity [9–12]. Smoking imparts a 1.6 to 2-fold increased risk for EAC and elevates BE risk [13–16]; whereas, reports on alcohol are mixed, largely supporting it as a stronger risk factor for esophageal squamous cell carcinoma compared to EAC. Other non-modifiable factors linked to BE and EAC progression include male gender, hiatal hernia, Caucasian race and age [3, 17, 18]. In terms of modifiable risk reducing factors, plant based diets rich in fruits, vegetables and fiber are associated with decreased risk for EAC and more animal based diets and red meat consumption with increased risk [19–24]. Although BE is the only precursor lesion for EAC, most BE patients are relatively healthy and only a small portion of BE lesions progress to EAC [25]; thus, identification of non-toxic cancer inhibitory agents, acceptable for long-term administration, are a logical focus. A number of bioactive constituents derived from plant-based sources have favorable toxicity profiles. Specifically, LBR have shown cancer inhibitory effects in a broad range of preclinical models of oral, esophageal, colon, breast and skin cancer [26–33] supporting the transition to clinical investigations in patients with cancer or at increased risk for cancer development [34]. We hypothesized the LBR may slow BE progression by protecting against GERD-induced aberrant signaling cascades, oxidative and nitrosative stress and subsequent DNA damage. A 6 month pilot study was conducted to investigate whether LRB inhibit oxidative damage and modulate markers associated with GERD and progression of Barrett's esophagus.

## RESULTS

### Characteristics of the Barrett's patient population, including changes in body mass index and cholesterol levels

**Figure 1:**
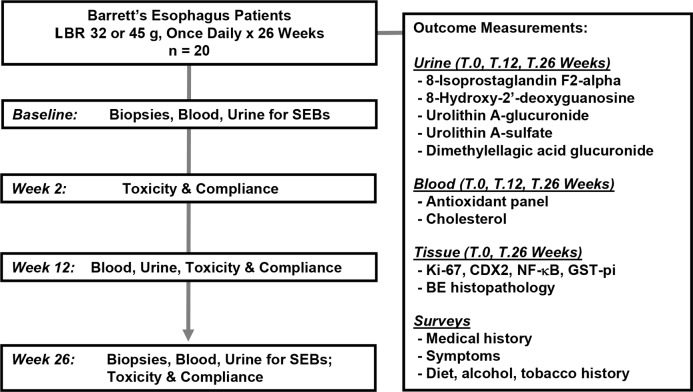
Study Schema

**Figure 2:**
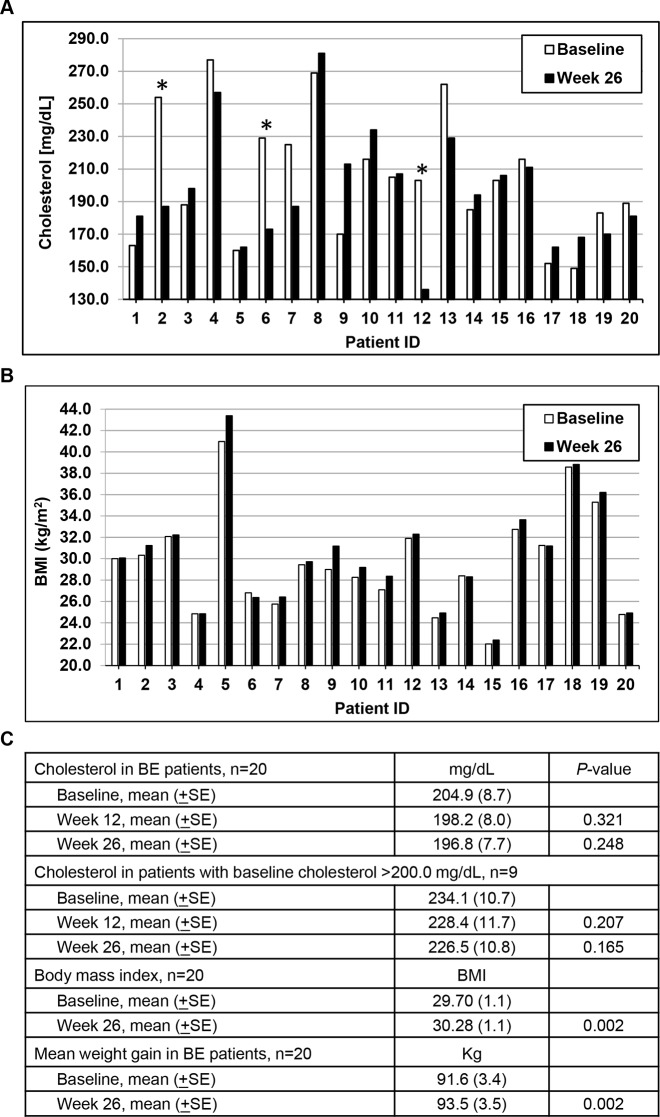
Changes in cholesterol and body mass index (BMI) levels over time **A.** Individual patient cholesterol levels at baseline and following 26 weeks of LBR treatment. ^*^Patients who initiated or changed cholesterol medication during the study. **B.** Individual patient BMI levels at baseline and following 26 weeks of study. **C.** Mean cholesterol levels (±SE) in all patients and in patients with elevated baseline cholesterol levels (excluding patients that changed or initiated cholesterol lowering medication use during the study). Mean BMI and mean weight gain (±SE) at baseline and at 26 weeks of study.

The study schema including outcome measurements are depicted in Figure 1. Patient demographics and characteristics of the study population that was consented, enrolled and completed the 6 month intervention with LBR are summarized in Table 1. Figure 2 details changes in body mass index (BMI), weight, and total cholesterol levels. A total of 20 enrolled patients completed the 6 month dietary intervention. One enrolled patient discontinued the study due to non-study related medical issues. The mean age of patients was 58.9 years (range, 44-74). All subjects were Caucasian and consisted of 16 males and 4 females, reflective of the population distribution of BE cases. Patients reported that they had symptoms of GERD for a mean of 18 years with 35% of patients still suffering from regular heartburn despite proton pump therapy. The average age of GERD onset among study subjects was 41.0 years (range, 13-60). At baseline, the average length of the Barrett's segment was 3.35 cm (range, 1.0–8.0) and following the 26-wk dietary intervention BE length was non-significantly lowered to 3.30 cm (range, 2.0–8.0). All enrolled patients had confirmed BE with intestinal metaplasia on one or more biopsies at baseline. Dysplasia was not detected in any of the patient biopsies at baseline or in any biopsies collected at 26 weeks of study.

**Table 1:** Characteristics of the patient population

Characteristic	
Age, mean years (±SE)	58.9 (2.2)
Range, years	44 - 74
Gender	
Female, n (%)	4 (20)
Male, n (%)	16 (80)
Education	
High school graduate, n (%)	20 (100)
College graduate, n (%)	10 (50)
Graduate degree, n (%)	6 (30)
GERD	
Age of onset, mean years (±SE)	41.0 (2.9)
Range, years	13 - 60
Mean years w/GERD (±SE)	18.0 (2.0)
Regular heartburn, n (%)	7 (35)
Endoscopy	
Metaplasia, n (%)	20 (100)
Esophagitis, n (%)	3 (15)
Hiatal hernia, n (%)	20 (100)
Length of Barrett's (cm)	
Baseline, mean (±SE), range	3.35 (0.4), 1-8 cm
Week 26, mean (±SE), range	3.30 (0.3), 2-8 cm
Exposures	
Baseline, Overweight, n (%)	7 (35)
Baseline, Obese, n (%)	9 (45)
Week 26, Overweight, n (%)	6 (30)
Week 26, Obese, n (%)	10 (50)
Current smoker, n (%)	3 (15)
Current smokeless tobacco use, n (%)	1 (0.05)
Past smoker, n (%)	8 (40)
Never tobacco, n (%)	8 (40)
Current beer, n (%)	17 (85)
Current wine, n (%)	15 (75)
Current liquor, n (%)	12 (60)
Never alcohol, n (%)	3 (15)

**Figure 3:**
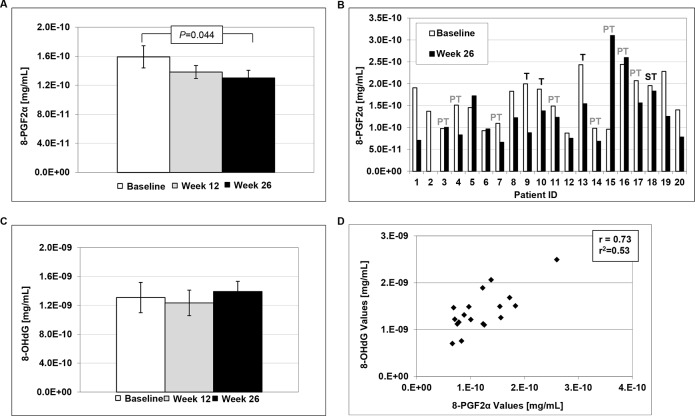
Urinary levels of 8-epi-prostaglandin F2α (8-PGF2α) and 8-hydroxy-2′-deoxyguanosine (8-OHdG) **A.** LBR induced significant decreases in 8-PGF2α levels at 26 weeks of study compared to baseline (Students *t-*test, *P*=0.044). **B.** Individual levels of 8-PGF2α at baseline and post-treatment. T=tobacco user, PT=past tobacco user and ST=smokeless tobacco user. **C.** Levels of 8-OHdG at Baseline, week 12 and week 26 of study. **D.** Correlation between 8-PGF2α and 8-OHdG levels at 26 weeks of study.

At study baseline 80% of the patients were overweight or obese based on BMI measurements and after the 6 month intervention 80% of patients remained with BMI ≥25. Mean patient BMI values significantly increased from 29.70 at baseline to 30.28 at 26 weeks of study (*P=*0.002, paired t-test, two tailed) as shown in Figure 3. On average, BE patients gained 1.8 kg (range, −1.4 to 4.2) during the 6 month intervention. Despite weight gains and increased BMI measurements, total cholesterol levels non-significantly declined following 12 and 26 weeks of the LBR intervention as shown on a per patient basis in Figure 2A and summarized in 2C. The magnitude of the reduction was greater in patients with cholesterol levels over 200 mg/dL (*P=*0.165). One patient changed cholesterol lowering medication during the study and two initiated use of cholesterol lowering medications; all are marked with an asterisk and were excluded from the cholesterol data presented in Figure 2C. Still, select patients (ID 4, 7, 13, 19) had marked reductions in total cholesterol levels which was not explained by medication use. Other exposure information collected focused on tobacco and alcohol use. Four patients were tobacco users, three smokers and one used smokeless tobacco products. In addition, 40% were past smokers and most reported consumption of beer (85%), wine (75%) or liquor (60%).

### Compliance and toxicity

Patients were contacted by telephone 2 weeks after study initiation and queried regarding potential toxicities associated with the intake of LBR and their history of consumption for the previous week. The study nurse coordinator recorded information on consumption compliance and any potential adverse events at week 12 and week 26 outpatient visits. Patients also kept a daily study calendar recording the time of berry consumption which was returned with all berry packets at study completion for an additional check on compliance. Overall, compliance was strong with 96.6% of patients consuming the berries as scheduled each morning (median 97.8%, range, 86.1-100.0). Berries were well tolerated with only three patients reporting grade 1 adverse side effects that were likely linked to berry consumption. Reported events included epigastric pain, diarrhea and constipation, but all were resolved and patients completed the study.

### LBR reduce urinary levels of 8-epi-prostaglandin F2α (8-PGF2α), but not 8-hydroxy-2′-deoxyguanosine (8-OHdG)

Consumption of LBR for 26 weeks significantly decreased urinary levels of 8-PGF2α, a marker of lipid peroxidation, as illustrated in Figure 3. Mean levels were reduced from 1.6E-10 at baseline to 1.4E-10 at week 12 and 1.3E-10 at week 26 (Figure 1A, *P*=0.044, *t*-test). Overall, 11 of 19 patients experienced marked reductions in 8-PGF2α following 26 weeks of LBR consumption as shown in Figure 2B and one patient a marked increase for reasons that are not clear (patient 15). In addition, levels of 8-PGF2α correlated with levels of 8-OHdG (Figure 1D), but mean levels of urinary 8-OHdG were not significantly altered following the 6 month intervention with LBR in BE patients. In addition, there was greater variability in levels of 8-OHdG.

### Consumption of LBR resulted in the formation of urinary ellagitannin metabolites in BE patients

Berries are rich in bioactive polyphenols including ellagitannins, but this appears to be the first report characterizing levels of ellagitannin metabolites in urine following treatment with LBR. At baseline or pretreatment urolithin A-glucuronide and urolithin A-sulfate levels were undetectable and only 15% of patients expressed low to moderate detectable levels of dimethylellagic acid glucuronide (DMEAG) suggesting low consumption of polyphenols prior to study initiation. Following 12 and 26 weeks of LBR consumption levels of all three metabolites significantly increased compared to baseline levels; however, the concentrations and pattern differed as detailed in Table 2 and Supplementary Table S1. Urolithin A-glucuronide was detected in urine of 15% of patients at baseline, but detected in 85% of patients following 12 and 26 weeks of LBR consumption. Also, urolithin A-glucuronide levels significantly increased from a mean of 65.9 ng/mL at baseline levels to 1287 ng/mL and 1723 ng/mL at 12 and 26 weeks of study, respectively. Urolithin A-sulfate levels were below detectable limits at baseline yet significantly increased to 114 and 119 ng/mL at 12 and 26 weeks, respectively. Urolithin A-sulfate was detected in urine of 60% of BE patients following LBR consumption at both 12 and 26 weeks of study. DMEAG was detected among more patients, 95% at 12 weeks and 70% at 26 weeks, but levels were considerably lower at week 26 (9 ng/mL) compared to week 12 (45 ng/mL) for reasons that are not related to LBR consumption. Therefore, DMEAG may be a less reliable biomarker of intake or compliance compared to urolithin A-glucuronide. All 26 week measurements were 24 to 32 hours following final consumption of LBR which has implications for rapidly cleared metabolites.

**Table 2:** Patients expressing ellagitannin urinary metabolites at baseline, 12 and 26 weeks of study

*Patients expressing metabolite*	Baseline n/total (%)	12 Weeks n/total (%)	26 Weeks n/total (%)
Urolithin A-Glucuronide	3/20 (15)	17/20^a^ (85)	17/20^a^ (85)
Urolithin A-Sulfate	0/20 (0)	12/20^a^ (60)	12/20^a^ (60)
Dimethylellagic Acid Glucuronide	0/20 (0)	19/20^a^ (95)	14/20^a^ (70)
***Metabolite***	**Baseline** **mean ng/mL (±SE)**	**12 Weeks** **mean ng/mL (±SE)**	**26 Weeks** **mean ng/mL (±SE)**
Urolithin A-Glucuronide	69.5 (43.9)	1286.6 (265.3)^b^	1723.0 (356.6)^b^
Urolithin A-Sulfate	Not detected	113.8 (27.1)^b^	118.7 (39.9)^b^
Dimethylellagic Acid Glucuronide	Not detected	45.0 (7.4)^b^	8.72 (2.2)^b^

^a^ Statistically significant difference from baseline population proportions as determined by Chi-square test (*P*<0.01).

^b^ Statistically significant difference from baseline levels as determined by Student's *t*-test, 2-sided, paired (*P*<0.01).

### Dietary intake and plasma antioxidant levels in BE patients

As shown in Table 3 a panel of 8 antioxidants, mainly carotenoids, was evaluated in the plasma of each patient at baseline and at 26 weeks of study to investigate whether LBR consumption had any impact on levels and also to determine whether non-LBR linked antioxidants may be altered as potential indicators of other dietary changes. In addition, a 12 page food frequency questionnaire was utilized to determine levels of approximately 125 dietary components, including specific nutrients as an additional assessment of dietary factors. Mean levels of retinol and zeaxanthin significantly increased when comparing levels at baseline to week 26. The food frequency questionnaire revealed significant declines in six of over 130 dietary variables when comparing baseline dietary intakes to those at 26 weeks of study. Significant reductions were noted for Vitamin E, total alpha-tocopherol equivalents, alpha-tocopherol, natural alpha-tocopherol, linoleic acid and behenic acid (*P*≤0.05, Students *t-*test, 2 tailed, paired). None of the other dietary variables were significantly changed from baseline levels to levels calculated at week 26 supporting that as a group few dietary changes occurred during the intervention period.

**Table 3:** Blood antioxidant levels in BE patients over time

Antioxidant	Baseline Mean (± SE) (ng/mL)	26 Weeks Mean (± SE) (ng/mL)	*P*-value
Retinol	599 (0.031)	641 (0.032)^a^	**0.021**
Retinyl Palmitate	25 (0.002)	23 (0.003)	0.911
Lutein	106 (0.012)	115 (0.011)	0.265
Zeaxanthin	36 (0.003)	48 (0.005)^a^	**0.002**
β-cryptoxanthin	45 (0.006)	46 (0.005)	0.912
Lycopene	228 (0.028)	229 (0.026)	0.977
α-carotene	46 (0.007)	40 (0.005)	0.441
β-carotene	178 (0.021)	185 (0.026)	0.457

^a^ Statistically significant difference from baseline levels as determined by Student's *t*-test, 2-sided, paired (*P*<0.05).

### Effects of LBRs on immunohistochemical markers in BE epithelium

**Figure 4:**
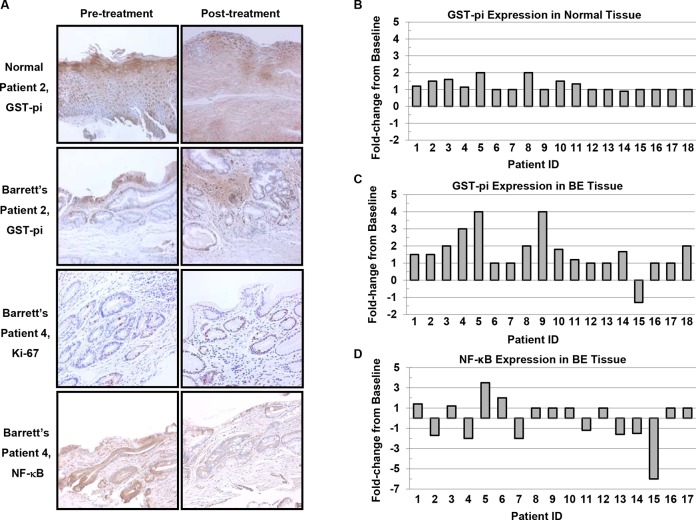
Immunohistochemical staining of normal esophageal epithelium and Barrett's epithelium (200X) **A.** Representative photomicrographs of pre-treatment and post-treatment esophageal biopsies stained for GST-pi, Ki-67 and NF-κB. **B.** Fold-change from Baseline in GST-pi levels in normal esophagus and **C.** Barrett's epithelium. **D.** Fold-change from baseline in NF-κB levels in Barrett's epithelium.

Figure 4A shows representative photomicrographs of GST-pi, Ki-67 and NF-κB stained esophageal biopsy specimens at baseline and after 26 weeks of the LBR intervention. Overall, mean levels of Ki-67 and NF-κB were not significantly altered following the intervention. Mean levels of NF-κB declined from 1.58 to 1.40 in BE tissues (*P*=0.234, paired *t*-test). On the individual level, marked down regulation of the transcription factor NF-κB (≥1.5-fold, Figure 4) occurred in 6 patients (35.3%) and increased levels were detected in 2 patients. Levels of the proliferation marker Ki-67 declined in 2 patients. Only low levels of CDX2 were expressed in baseline BE biopsies and not among all patients limiting the usefulness of this marker in the context of evaluating potential reductions. Mean levels of GST-pi significantly increased from 0.89 to 1.46 (64%) in BE biopsies post-intervention (*P*=0.002), with individual level fold changes illustrated in Figure 4B and 4C and summarized in Supplementary Table S2. Increased levels of GST-pi staining occurred in 55.6% or 10 of 18 evaluable paired patient biopsies post intervention. Only one patient (15, a past tobacco user) presented with a marked decline in GST-pi staining at week 26. Although mean levels of GST-pi staining were not significantly increased in normal epithelial biopsies, ≥1.5-fold increased levels occurred in the normal epithelium of a patient subset involving 5 of 18 patients.

### Factors impacting symptoms of gastroesophageal disease at baseline and at 26 weeks of study

Patients were queried at before and after 26 weeks of LBR administration regarding factors they perceived to improve or worsen their GERD symptoms as shown in Table 4. Major factors reported to increase symptoms of GERD included eating large meals or spicy meals as well as acidic foods, followed by stress, consuming citrus or rich foods, eating out, eating near bedtime and eating fried or fatty foods. Caffeine as a trigger was reported by only 20% of patients to worsen GERD symptoms and it was the only factor that significantly declined post berry intervention. Top factors reported to decrease GERD symptoms included medication compliance, dietary modifications, eating small or bland meals, sleeping on an incline, relaxing and eating late. Consumption of red meat, fiber, fruits and vegetables or exercising had little to no perceived impact on GERD.

**Table 4:** Factors reported to impact symptoms of GERD at baseline and 26 weeks of study

Increase GERD	Baseline %	26 Weeks %	Decrease GERD	Baseline %	26 Weeks %
Large meal	50	40	Medication compliance	100	100
Spicy meal	50	40	Dietary modifications	65	50
Acidic foods	50	70	Small meals	60	60
Stress	45	35	Sleeping on incline	40	40
Citrus foods	35	25	Bland diet	40	30
Rich foods/dessert	35	35	Relaxing	35	35
Eating out	30	25	Avoid eating late	30	30
Eating near bedtime	30	30	Fruits & vegetables	10	5
Fried or fatty foods	25	25	Exercise	5	5
Caffeine	20	10^a^	Fiber	0	0
Alcohol	20	5			
Chocolate	20	25			
Exercise	10	10			
Red meat	0	10			

^a^ Statistically significant difference from baseline population as determined by Chi-square test (*P*<0.05)

## DISCUSSION

Many BE patients are successfully treated to relieve symptoms associated with GERD; however, curative therapies or those proven inhibitory against EAC development remain elusive. Thus, we conducted a phase 1 pilot study investigating the ability of LBR to mitigate GERD-induced aberrant signaling cascades and subsequent oxidative and DNA damage which is linked to cancer progression.

LBR were well tolerated when administered up to 45 g daily for a period of 6 months. Adverse events potentially linked to berry consumption were reported by 15% of patients and included epigastric pain, diarrhea and constipation. Patients experiencing one or more of these events were advised to reduce the amount of LBR consumed for a period 1-4 days and then resume the full amount which readily resolved the grade 1 toxicities reported and permitted all patients to remain on study. Patient compliance was high with 96.6% consuming the berries as scheduled each morning. As previously described, LBR are rich in a number of nutrients and bioactive polyphenols, including ellagic acid [35]. Ellagic acid is converted to dimethylellagic acid glucuronide (DMEAG) and urolithins following consumption of pomegranate juice [36], but has not been documented following LBR consumption. Thus, as a potential additional marker of compliance we characterized levels of DMEAG and urolithin metabolites produced by the gut microbiota from ellagic acid (EA) pretreatment and following 12 and 26 weeks of LBR consumption. At baseline 15% of BE patients expressed detectable levels of Urolithin A-glucuronide, but none expressed detectable levels of Urolithin A-sulfate or DMEAG. Levels of DMEAG and both urolithins significantly increased following 12 and 26 weeks of LBR administration; however, the magnitude of effect and consistency was strongest for Urolithin A-glucuronide levels. Thus, measuring specific berry metabolites may serve as an additional indicator of LBR compliance for clinical evaluations with the caveat that the time from last berry consumption, individual level differences in metabolic machinery, and microbiome profiles will likely impact results. A recent clinical trial in targeting colon cancer patients with LBR (60 g daily) utilized a non-targeted metabolic analysis to uncover 34 and 16 metabolites in patient urine and blood, respectively following 1-9 weeks of LBR administration [37]. The latter research illustrates that even short-term LBR treatment alters metabolism of amino acids, lipids and xenobiotics.

Plasma antioxidant levels and dietary intake information was collected on each patient to account for possible changes in dietary patterns which might influence study results given investigation of a food-based product. In addition, low dietary antioxidant intake has been linked to the development of BE and increased risk of EAC [17, 38]; whereas, dietary anthocyanidin consumption is inversely associated with BE [39]. Thus, anthocyanidin rich food sources, such as berries, may offer benefits to patients with BE or those at risk for EAC progression. On a population basis there were no significant changes in consumption of fruits and vegetables from baseline to study completion. Over the course of the intervention, significant differences were detected in only 6 of over 130 dietary measures. Four dietary variables, related to vitamin E, significantly declined as did levels of linoleic and behenic acids. Specifically, total vitamin E, total alpha-tocopherol and natural forms of alpha-tocopherol were significantly reduced at week 26 compared to baseline levels. Major dietary sources of vitamin E include vegetable oils, nuts, seeds, whole grains, and green leafy vegetables [40]. Linoleic acid is the main polyunsaturated fat in vegetable oil and is also found in appreciable levels in nuts, seeds and processed foods due to the contribution of soybean, corn, safflower or canola oils [41]. Behenic acid is a major component of Ben oil and is also present in other commonly ingested oils, including canola and peanut. Moreover, behenic acid is reportedly a cholesterol raising saturated fatty acid in humans [42]. Thus, we cannot rule out a change from baseline to 26 weeks of study in terms of consumption of vegetable oils based on their dietary abundance and because they are a source for each dietary variable altered. Results from a serum antioxidant panel, comprised of 8 carotenoids, showed that both retinol and zeaxanthin significantly increased at week 26 compared to baseline population levels. A number of carotenoids have been detected in raspberries, including zeaxanthin which may contribute to increased blood levels in BE patients post-intervention. However, most retinol or preformed vitamin A is found in animal products; thus, not likely due to LBR consumption. Plant based beta-carotene can be cleaved into retinol in the body, but beta-carotene levels were not significantly altered over the study duration making this an unlikely contributor. Individual patient level data supports that marked increases in retinol were limited to four patients, two of which experienced large weight gains (Patient 5 and 9) and increased BMIs over the 6 month intervention. Overall, the FFQ data supported very limited dietary changes outside of the intervention.

In recent years obesity has emerged as a risk factor for GERD, BE and EAC. It is concerning that 80% of BE patients were obese or overweight at study baseline and that mean weight significantly increased by 1.8 kg during the intervention period which in turn increased the proportion of obese patients (BMI scores ≥ 30) to 50%. Despite significantly increased weight gains and BMIs, total cholesterol levels were non-significantly lowered (*P=*0.165) following 26 weeks of LBR treatment, particularly in patients with elevated baseline cholesterol levels. Three patients with elevated cholesterol levels at baseline changed or initiated use of cholesterol lowering medications between 8 and 12 weeks of study. They were excluded (Patients 2, 6, 12) from the cholesterol analysis as they would have resulted in a significant, but pharmacologically induced reduction in total cholesterol. In recent years there has been increasing interest and emerging evidence from intervention studies supporting that various berries impact cardiovascular risk factors [43]. Although the current study was not designed to address impact on CVD risk factors, results hint at positive effects on total cholesterol levels. These results are in alignment with recent studies evaluating the effects of black raspberry on lipid profiles, vascular endothelial function and blood pressure levels in patients that are prehypertensive or with metabolic syndrome [44, 45]. In addition, a number of the mechanisms mitigating cardiometabolic risk are hypothesized to reduce cancer risk, especially for inflammatory linked cancers.

Specific cancer-associated biomarkers were chosen based on knowledge that GERD remains the strongest risk factor for BE and EAC and is known to be linked to increased Inflammation, oxidative stress, aberrant activation of transcription factors and reduced antioxidant defence mechanisms. Specifically, at the tissue level markers of proliferation, detoxification, and inflammation-linked transcription factors with a focus on those altered early in the conversion of the normal squamous epithelium to metaplastic Barrett's epithelium were included. The intestine specific transcription factors CDX1 and CDX2 are reportedly triggers for metaplastic transition, with differential expression in normal esophageal mucosa compared to metaplasia and CDX2 expression increases with esophageal inflammation [46]. However, we detected only low levels of CDX2 expression in a subset of baseline BE patients limiting the usefulness of this marker for assessing chemopreventive effects. Changes in cellular proliferation were investigated via immunohistochemical staining for Ki-67, a nuclear protein increased in proliferating cells, especially those in G2, M, and latter half of the S phase. Overall, LBR treatment did not significantly alter the mean percent positive Ki-67 labelling index post-treatment. An earlier intervention in BE patients focusing on increasing fruit and vegetable consumption while reducing fat intake and body weight similarly reported no shift in cell proliferation post intervention despite a 35% increase in fruit and vegetable intake [47]. The authors proposed that hyper-proliferation may be a property of genetically abnormal BE metaplasia that cannot be reversed with dietary intervention, but that earlier changes linked to neoplasia, such as oxidative DNA damage or telomere length may be affected. A lack of effect on Ki-67 levels in the current study may also be due to insufficient concentrations of LBR reaching the target tissue or the need to administer LBR more frequently than once daily. 32 and 45 g of LBR was chosen based on preclinical results and to remain in a range that is behaviourally achievable for patients outside a clinical intervention. Two other pilot studies utilizing black raspberries to target colon cancer and familial adenomatous polyposis (FAP), respectively report reductions in proliferation levels measured by Ki-67 staining when LBR administration occurred 3 times daily (60 g total) or as a rectal suppository to FAP patients, allowing for relative direct delivery [48, 49]. In the colorectal trial select plasma cytokines, GM-CSF and IL-8, were significantly altered in patients consuming berries for more than 10 days and changes correlated with tissue level apoptosis induction and a reduction in proliferation [50]. These results support that LBR induce changes in circulatory markers rapidly (10 days), but that tissue specific alterations in proliferation and cell death require a longer duration of LBR administration (4 or more weeks) or potentially a higher cumulative dose. Alternatively, the administration of berry powder in an oil-based vehicle rather than water might permit more optimal adsorption of berry compounds into BE lesions.

Chronic reflux of bile salts, especially at acidic pH, induces inflammation and increases reactive oxygen and nitrogen species contributing to oxidative stress and progression to cancer. Elevated ROS levels have been detected in BE patients [51] where inflammatory driven oxidative stress contributes to an imbalance between the generation of damaging reactive electrophiles and antioxidant defense systems. Glutathione in conjunction with glutathione peroxidase and GST-pi enzymes are key for cellular detoxification and protecting macromolecules from attack [52]. GST-pi is the main GST enzyme expressed in the esophagus [53] and levels are reduced in BE compared to normal epithelium and as the histologic grade worsens toward EAC [54, 55]. GST-pi staining in BE epithelium significantly increased following LBR treatment compared to baseline levels with markedly increased levels among 55.6% of patients (10 of 18 evaluable paired patient biopsies). One patient who was a past smoker presented with decreased levels of GST-pi following the intervention, but overall results were favorable. Down-regulation of GST-pi in esophageal squamous cell carcinoma is associated with poor prognosis [56]. Thus, LBR upregulation of GST-pi is encouraging as it is associated with a normal esophageal phenotype and may serve to increase detoxification of injurious reflux components and ultimately mitigate BE progression.

NF-κB is a critical regulator of genes involved in multiple cancer associated processes including aberrant proliferation, altered immune response, apoptosis suppression, and stimulation of inflammatory pathways. This proinflammatory transcription factor is known to be upregulated by bile acid and to play a key role in the pathogenesis of EAC [57, 58]. In addition, NF-κB is associated with poor therapeutic response [57, 58]. Thus, targeting NF-κB activation is attractive for prevention as well as treatment. Mean tissue levels of NF-κB were not significantly altered following 26 weeks of LBR treatment; however, a subpopulation (35%) of BE patients responded with reduced levels of NF-κB post-treatment. Two patients experienced increased NF-κB levels at study completion. Evidence of LBR responders and non-responders has been reported in clinical trials targeting oral premalignancy as well [59, 60, 61]. Mallery and colleagues recently reported that 76.2% of patients treated with berry gel experienced reductions in premalignant lesion size, 41% improved lesion grade and that these responders demonstrated higher pretreatment levels of keratinocyte differentiation and berry metabolizing enzymes [61]. These results support inter-individual differences in metabolic and differentiation capacities impact responsiveness to berries and may offer a way to screen future patients for personalized intervention studies and to increase response efficacy.

Chronic exposure of the lower esophagus to a mixture of bile salts and stomach acid is well documented to induce inflammation, mucosal injury, oxidative stress and DNA damage contributing to BE and progression to EAC [62, 63]. Two additional markers, 8-PGF2α and 8-OHdG, were included as measures of lipid peroxidation and DNA damage, respectively. Both markers have been positively correlated to oxidative stress. Urinary excretion of 8-PGF2α was significantly reduced at week 26 compared to pretreatment levels, supporting that LBR modulated lipid peroxidation in 58% of the BE patients enrolled. 8-PGF2α is linked to oxidative stress and free radical damage and although levels correlated with levels of 8-OHdG; overall, mean levels of this DNA damage linked marker were not altered by LBR treatment. A recent study evaluating biomarkers of inflammation and oxidative stress in a BE cohort report that increased systemic levels of C-reactive protein and to lesser extent IL-6 are associated with progression to EAC supporting the inclusion of these two markers for evaluation in future trials evaluating anti-inflammatory agents conducted in BE patients [64]. To date, evaluations of pure supplements and antioxidants as cancer inhibitors in the clinical setting have generally been disappointing, especially in nutritionally sufficient populations. Evaluation of food-based inhibitors in a “whole” or natural form offers the advantage of delivering complex mixtures of inhibitory components which in turn holds the potential to mitigate multiple cancer processes. Adherence to the World Cancer Research Fund (WCRF) guidelines on lifestyle and cancer has recently been identified as a significant and independent protective factor against BE progression to EAC (OR 0.51, 95% CI 0.37-0.67) [65]. Specifically, disease progression is associated with reduced adherence to guidelines on fruit consumption, processed meat consumption, physical activity and sedentary habits. Hence, the report supports that positive dietary and lifestyle changes can modulate progression to EAC; the extent to which targeted food-based preventive agents can contribute is still being assessed. In the clinical setting, positive effects of black raspberries have been reported on preneoplastic lesions or cancers of the head and neck, esophagus, and colon [35, 48–50, 59–61]. Direct delivery of a black raspberry gel to lesions in the oral cavity results in histologic regression of oral intraepithelial neoplasia, improves histologic grade in a patient sub-set and significantly reduces LOH at tumor suppressor gene loci, modulates genes linked to RNA processing and growth factor recycling. In the colon, positive effects of LBR include anti-proliferative effects, activation of pro-cell death pathways, demethylation of tumor suppressor genes and improvements of plasma cytokine profiles. Similarly, research in obese non-cancer patients report reductions in serum cytokine levels and lipid profiles including IL-6, TNF-α, and cholesterol [44]. Impressively delivery of black raspberries as a suppository to FAP patients resulted in significant inhibition of FAP-associated polyp progression [49]. Herein, we describe that dietary administration of LBR to BE patients, significantly increased levels of GST-pi in BE epithelium, altered NF-κB in a subpopulation, significantly decreased urinary levels of 8-PGF2α, a marker of lipid peroxidation and global oxidative stress. We also report for the first time that LBR significantly increased levels of ellagitannin metabolites which may prove informative as a measure of compliance in future clinical investigations. Results from clinical investigations utilizing black raspberries have not all been uniform. Most of the research supports that a percentage of patients respond and others within the cohort do not, likely reflecting the heterogeneity between subjects and the complexity of each cancer or precursor lesion. Divergent outcomes may also be linked to differences in the mode, concentration, frequency and duration of black raspberry delivery. Research targeting sites which permit direct delivery, such as the oral cavity, has the advantage of requiring less product, increasing direct contact time with the lesion and takes advantage of local metabolism. Whereas, delivery of the suspended LBR powder described herein allowed for only passing contact with the tissue of interest, but likely resulted in greater systemic benefits due to the larger amount of LBR consumed. Still, common themes across studies have emerged including that LBR induce anti-inflammatory effects, reduce oxidative stress, impact metabolism and restore tumor suppressive activity. Although the optimum dose, duration and mode of berry delivery for cancer inhibition remains to be fully elucidated results to date suggest short durations and lower concentrations favorably impact lipid profiles, but potentially higher concentrations and longer durations of exposure are required for tissue specific effects. Further research is warranted to increase our knowledge of the cancer inhibitory mechanisms of berries and to improve our understanding of which patient populations are most likely to benefit.

## MATERIALS AND METHODS

### Clinical trial protocol

A schema of the study design and outcome measurements is shown in Figure 1. This phase I pilot study sought to investigate the effect of oral freeze-dried black raspberries on urinary metabolites and markers of lipid peroxidation, DNA damage and tissue markers of cellular proliferation, detoxification, and inflammation. In addition, a panel of carotenoids, as well as total cholesterol was measured from patient blood samples pre and post-berry administration. Twenty patients with a history of BE were recruited from The Ohio State University gastroenterology clinic. Patients were biopsied at baseline and those that met the eligibility criteria were randomized to take a slurry of 32 or 45 g of freeze-dried black raspberry powder in water once daily for 26 weeks. Patients were contacted at weeks 2, 12, and 26 to evaluate toxicity and compliance. Tissue biopsies were collected a baseline and at week 26 following berry treatment. Blood and urine were collected at baseline and weeks 12 and 26. Blood measurements included total cholesterol and an 8-marker antioxidant panel. Urine was utilized for assessing berry metabolites, and markers of DNA damage and lipid peroxidation. Each patient served as their own control allowing for comparisons before and after berry treatment on a per patient basis. All subjects remained on proton pump inhibitors or other medications they may have been taking throughout the study.

### Patient population and eligibility

Eligibility criteria included age 18 years or older, willingness to provide a complete medical history, a physical exam, complete a food-frequency questionnaire, positive endoscopy for BE (columnar lined esophagus, with specialized intestinal metaplasia) extending ≥1 cm above the gastroesophageal junction on the prescreening biopsy and on two previous biopsies, no history of invasive cancer within the past 5 years, normal organ function, normal serum chemistries, and signed informed consent approved by the Institutional Investigational Review Board. Exclusion criteria included any severe chronic or life-threatening diseases within 5 years, inability to return for scheduled follow-up visits, abnormal wound healing, esophageal varices or a history of varices or variceal bleeding, BE with high-grade dysplasia, coagulopathy that precedes taking esophageal biopsies safely, and excessive use of multivitamins or micronutrient supplements daily. This study was conducted in compliance with the protocol, the Institutional Review Board (IRB), the Code of Federal Regulations, and International Conference on Harmonization/Good Clinical Practice Guidelines. Written IRB approval was obtained prior to initiating the study.

### Berry preparation, route and schedule of administration

Fresh frozen black raspberries of the Jewel variety were purchased from the Stokes Fruit Farm (Wilmington, OH) and freeze-dried by Van Drunen Farms (Momence, IL) as previously reported [35]. In brief, powdered lyophilized berries were sealed into individual bags and distributed to the patients on a rolling basis. Eligible patients who signed an informed consent were instructed to store seven packets of berries in the refrigerator during the week they were scheduled to be consumed, and all other berries were stored in the freezer. Patients mixed the LBRs (32 g for females and 45 g for males) with about 170 ml of water and orally consumed the LBRs each morning at time of their choosing for 26 week study. The quantity of berries was approximated based upon targeting 5% berries in the diet considering average body weight figures and corresponding average caloric consumption requirements. This gram amount of lyophilized berries is equivalent to about 5 servings of fresh berries. Patients recorded the date and time of daily berry consumption on a study calendar and returned all empty berry packages as well as bags of berries which were not consumed. Compliance was determined based upon returned berry packages and further explored through metabolite analysis. Determination of potential chemopreventive substances, including vitamins, minerals, and select bioactive constituents were analyzed and previously reported [4, 35].

### Urine collection, storage and analysis for 8-OHdG, 8-PGF2α, and ellagitannin metabolites

Each patient collected urine for a 3-h period of time in the morning at study baseline or pre-treatment, at 12 weeks, and at 26 weeks or study completion. Urine was used to analyze biomarkers of oxidative DNA damage, lipid peroxidation and specific berry metabolites. Urine was stored at −80°C without preservatives. Replicates of three undiluted urine samples were shipped to Genox Corporation (Baltimore, MD) for analysis of 8-hydroxy-2′-deoxyguanosine (8-OHdG) and 8-epi-prostaglandin F2α (8-PGF2α) using immunoaffinity chromatography-monoclonal antibody–based enzyme-linked immunosorbent assays (ELISAs) as previously described (43,44). ELISA kits utilized for detection of 8-OHdG and 8-PGF2α were catalog no. KOG-200SE (Genox) and catalog no. 900-010 (Cayman Chemical, Ann Arbor, MI), respectively. Urinary creatinine levels were determined by using the Cayman Chemical creatinine assay (catalog no. 500701) according to manufacturer instructions. To control for potential differences in patient urine volume, levels of urinary 8-OHdG and 8-PGF2α were divided by levels of urinary creatinine. Analysis of urinary ellagitannin metabolites were conducted utilizing previously published methods [36]. Briefly, 10 mL aliquots of urine were diluted with H_2_O (2% formic acid):MeOH (9:1, v:v), vortexed, centrifuged and the supernatant filtered prior to LC-MS/MS analysis for determination of urolithin A-glucuronide, urolithin A-sulfate and dimethylellagic acid glucuronide.

### Dietary intake estimates

Each patient filled out a dietary Food Frequency Questionnaire (FFQ) reporting the frequency of consumption and portion size of a series of foods or beverages, as well as information on food preparation, to allow for more refined estimates of nutrient intake. All FFQs were processed at the Fred Hutchinson Cancer Research Center Shared Resource for Nutrition Assessment. Nutrition Assessment at Fred Hutchinson uses the University of Minnesota Nutrition Data Systems for Research software for data entry and nutrient analysis stemming from the 12 page FFQ. The FFQ information generated intake estimates for approximately 150 nutrients or nutrient constituents and were utilized to evaluate whether patients experienced significant dietary changes during the study.

### Blood antioxidant levels

Plasma was collected from each patient pretreatment and at 12 and 26 weeks of study. A panel of 8 antioxidants, largely carotenoids (retinol, retinyl palmitate, lutein, zeaxanthin, β-cryptoxanthin, lycopene, α-carotene, β-carotene), were assessed in each patient at baseline and following 26 weeks of berry consumption by Genox Corporation, Baltimore, MD. Total cholesterol was also determined as part of a routine testing at OSU hospitals at each time-point. These measurements served as a complement to FFQ intake information, further reflecting dietary habits.

### Factors impacting GERD symptoms at baseline and at 26 weeks of study

Based on factors reported in the literature, each patient was asked whether specific factors increased or decreased their symptoms of GERD at study baseline and again at study completion or 26 weeks of study as summarized in Table 4.

### Biopsy collection and pathology assessment

Four-quadrant BE biopsy specimens were obtained every other cm starting 1 cm above the GE junction. In addition, two specimens each of normal esophagus, cardia and fundus were collected. Biopsies were fixed in 10% NBF for 24 hours (± 2 hours), changed into PBS and stored refrigerated until routinely processed and paraffin embedded. A total of 50 (4-*μ*m) serial sections were cut resulting in 25 slides per biopsy specimen. Every fifth slide was H&E stained and evaluated by a single pathologist (W.F.) and slides selected for immuno-staining.

### Immunohistochemical staining and image analysis

Tissues were antigen retrieved by microwaving in 10 mM sodium citrate (pH 6.0) for 12 minutes for Ki-67 and 4 minutes (pH 6.0) for NF-κB and GST-pi staining. Tissues were blocked with 3% H_2_O_2_ for 20 min, casein for 15 min, goat serum for 20 min, and avidin/biotin for 30 min. Slides were incubated with the primary antibodies Ki-67 (Vector Laboratories, Burlingame, CA.; 1:120, 1 hour), NF-κB (p50/p105, Santa Cruz Biotechnology, Inc, 1:120, 2 hours) and GST-pi (Lab Vision Corporation, Fremont, CA; 1:150, 2 hours). Human-adsorbed link (biotinylated anti-immunoglobulin) followed for 20 min, streptavidin-horseradish peroxidase label for 20 min, and a final incubation with 3,3′-diaminobenzidine for 3.5 min to permit biomarker visualization. Slides were counterstained with hematoxylin, dehydrated in graded alcohols and cover-slipped with Permount. Positive (breast carcinoma and esophageal adenocarcinoma) and negative controls (mouse antiserum) were included in staining runs. Immunohistochemical stained slides were viewed at 100X with a Nikon bright-field microscope mounted with a high-resolution spot camera interfaced with a computer containing Image-Pro Plus image analysis software (Media Cybernetics, Rockville, MD). Briefly, images were grabbed, color thresholds set permitting quantification of positive brown stained areas, followed by threshold establishment for both positive (brown) and negative (blue) stained areas (Figure 4) allowing calculation of the mean labeling index for nuclear staining. Cytoplasmic staining was quantified utilizing a 0 to +3 scale as previously described [66].

### Statistical analysis

Student's t-test (two-tailed, paired) was used to evaluate the effect of LBRs treatment on mean levels of urinary 8-OHdG, 8-Iso-PGF2 levels, urolithins, DMEAG and to analyze changes in body mass index (BMI) measurements at Week 26 compared with baseline. Correlation analysis was conducted to derive a correlation coefficient value for evaluating the relationship between the two urinary markers of oxidative stress at 26 weeks of study. Chi-square test was used for assessing changes in number of patients expressing elevated metabolites over time as well as for factors increasing or decreasing symptoms of GERD pre to post-treatment.

### Toxicity and compliance evaluation

The clinical nurse coordinator contacted enrolled patients by telephone 2 weeks following study initiation and queried each patient regarding potential adverse events or toxicities associated with the intake of LBRs. Subjects were also queried at their Week 12 and Week 26 outpatient visits regarding any potential adverse events. In addition, at 2, 12, and 26 weeks of study, detailed information was recorded regarding medication and supplement use as well as berry consumption compliance for the previous week. Patients also kept a daily compliance calendar with a record of the time the berry packet was consumed. All empty and all un-opened packets were returned and counted as an additional verification on compliance.

## SUPPLEMENTARY MATERIALS TABLES


