# Supplementary material for: A phase I pilot study evaluating the beneficial effects of black raspberries in patients with Barrett’s esophagus
Source: Oncotarget. 2016 Jul 7;9(82):35356–72. doi: 10.18632/oncotarget.10457 (PMC6219678; doi:10.18632/oncotarget.10457)
Supplement: Supplementary file 1 [file oncotarget-09-35356-s001.pdf]

A phase I pilot study evaluating the beneficial effects of black raspberries in patients with Barrett's esophagus

SUPPLEMENTARY TABLES

Supplementary Table S1: Individual level patient compliance and urinary levels of ellagitannin metabolites at baseline, 12 and 26 weeks of study

Pat ID	LBR Compliance (%)	Urolithin A-Glucuronide (ng/mL)			Urolithin A-Sulfate (ng/mL)			Dimethylellagic Acid Glucuronide (ng/mL)		
		Week 0	Week 12	Week 26	Week 0	Week 12	Week 26	Week 0	Week 12	Week 26
1	90.6	0	0	0	0	0	0	0	24	5.7
2	91.1	0	280	1500	0	0	0	0	112	28
3	99.4	0	162	50	0	0	0	0	125	0
4	100	0	1920	2460	0	160	280	0	16	0
5	100	810	1020	2880	0	96	170	0	72	8.2
6	96.6	0	1720	590	0	290	96	0	81	10
7	100	340	4460	4020	0	360	90	0	55	18
8	94.2	0	2760	2660	0	160	104	0	42	8.4
9	100	0	2540	2570	0	215	84	0	27	0
10	86.1	0	320	110	0	0	0	0	29	0
11	99.4	0	0	0	0	0	0	0	67	5.8
12	96.7	0	1440	1620	0	104	76	0	47	5.1
13	98.9	0	0	0	0	0	0	0	49	16
14	97.3	240	1580	3950	0	87	280	0	37	8.7
15	96.2	0	1640	1290	0	304	115	0	25	6.8
16	97.3	0	1920	4940	0	84	608	0	48	36
17	98.4	0	0	0	0	0	0	0	13	5.6
18	99.5	0	2400	1690	0	288	36	0	0	0
19	91.8	0	610	450	0	0	0	0	12	0
20	98.4	0	960	3680	0	128	435	0	18	12

Note: LBR=lyophilized black raspberry powder.

Supplementary Table S2: Summary of individual patient level changes in urinary, blood and tissue biomarkers following 26 weeks of LBR treatment

Pat ID	Tobacco Use	Weight (kg, change)	8-Iso-Pros (change)	8-OHdG (change)	GSTpi (fold-change)	NF-κB (fold-change)	Zeaxanthin (change)	Uro A-Gluc (change)	Uro A-Sulf (change)	DMEAG (change)
1	Never	0.2	decrease	no change	1.5	1.4				+
2	Never	2.0	ND	ND	1.5	-1.7		+		+
3	Past Smoker	0.5	no change	increase	2.0	1.2		+		
4	Past Smoker	0.0	decrease	decrease	3.0	-2.0	+	+	+	
5	Never	6.6	no change	increase	4.0	3.5	+	+	+	+
6	Never	-1.4	no change	no change	1.0	2.0		+	+	+
7	Past Smoker	2.3	decrease	decrease	1.0	-2.0		+	+	+
8	Never	0.9	decrease	decrease	2.0	1.0		+	+	+
9	Smoker	6.8	decrease	decrease	4.0	1.0	+	+	+	
10	Smoker	3.2	no change	increase	1.8	1.0		+		
11	Past Smoker	4.2	decrease	decrease	1.2	-1.2				+
12	Never	1.2	no change	increase	1.0	1.0		+	+	+
13	Smoker	1.5	decrease	increase	1.0	-1.6		+		+
14	Past Smoker	-0.3	decrease	increase	1.7	-1.5	+	+	+	+
15	Past Smoker	1.1	increase	increase	-1.3	-6.0		+	+	+
16	Past Smoker	2.5	no change	increase	1.0	1.0		+	+	+
17	Past Smoker & Smokeless	-0.2	decrease	no change	1.0	1.0				+
18	Smokeless	0.8	no change	increase	2.0	-10		+	+	
19	Never	2.9	decrease	decrease	ND	ND		+		
20	Never	0.5	decrease	no change	ND	ND		+	+	+

Note: LBR=lyophilized black raspberries. Change indicates the change of the variable at week 26 of study compared to baseline.
